# Identification of two novel mutations in the *GALNT3* gene in a Chinese family with hyperphosphatemic familial tumoral calcinosis

**DOI:** 10.1038/boneres.2016.38

**Published:** 2016-11-08

**Authors:** Lihao Sun, Lin Zhao, Lianjun Du, Peipei Zhang, Minjia Zhang, Min Li, Tingting Liu, Lei Ye, Bei Tao, Hongyan Zhao, Jianmin Liu, Xiaoyi Ding

**Affiliations:** 1 Department of Endocrine and Metabolic Diseases, Rui-jin Hospital, Shanghai Jiao-Tong University School of Medicine, 197 Rui-jin Er Road, Shanghai 200025, China; 2 Department of Endocrinology and Metabolism, Zhong-shan Hospital, Fudan University, Shanghai 200032, China; 3 Department of Radiology, Rui-jin Hospital, Shanghai Jiao-Tong University School of Medicine, 197 Rui-jin Er Road, Shanghai 200025, China

## Abstract

Hyperphosphatemic familial tumoral calcinosis (HFTC) is a rare, autosomal recessive genetic disease. This disease is characterized by the progressive calcification of soft tissues leading to symptoms of pressure and hyperphosphatemia but normal concentrations of serum calcium with or without an elevation of 1,25-dihydroxyvitamin D_3_ levels.HFTC is caused by loss-of-function mutations in the *GALNT3*, *FGF23* or *KL* genes. Here, we identified two novel mutations in the *GALNT3* gene in a Chinese family with HFTC. Identification of a novel genotype in HFTC provides clues for understanding the phenotype–genotype relationships in HFTC and may assist not only in the clinical diagnosis of HFTC but also in the interpretation of the genetic information used for prenatal diagnosis and genetic counseling.

## Introduction

Familial tumoral calcinosis (FTC) is a rare, autosomal recessive genetic disease. The main clinical manifestation of FTC is the development of calcified masses predominantly at peri-articular locations, especially the hips, leading to intolerable pain, skin ulcerations and secondary skin and bone infections.^1^

According to serum phosphate concentrations, FTC can be classified as hyperphosphatemic (HFTC) or normophosphatemic FTC.^2^ FTC is caused by a loss-of-function mutation in one of the following genes: *FGF23,* which encodes the potent phosphaturic protein fibroblast growth factor 23; *GALNT3*, which encodes the uridine diphosphate-N-acetyl-α-D-galactosamine-polypeptide N-acetylgalactosaminyl-transferase 3 (GalNAc-T3), a glycosyl transferase responsible for FGF23 *O*-glycosylation and proper secretion; or KL, which encodes Klotho, an FGF23 co-factor required to activate FGF receptors.^3–7^

The incidences of FTC in men and women are similar; however, the incidence in black patients of African descent is relatively high, followed by that in white patients from the Middle East.^1^ Only one observed individual with HFTC caused by an *FGF23* mutation was Japanese.^7,8^ Herein, we report two HFTC cases with two novel mutations in the *GALNT3* gene.

## Materials and methods

### Case report

The proband of this study was a 13-year-old boy. When he was 2 years old, his parents accidentally discovered hard masses with slight tenderness on his right hip and feet. With increasing age, the masses increased in size. Several operations were performed to remove the masses on his feet and right hip.

**Figure 1:**
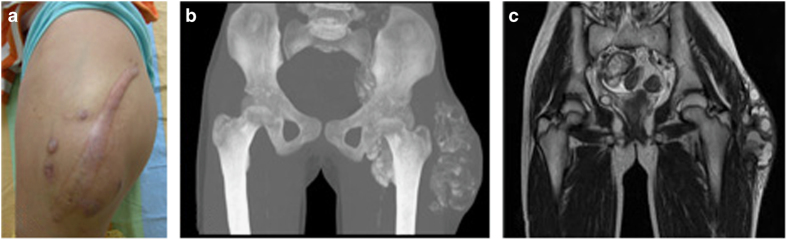
Imaging data for the sister of the patient. (**a**) The left hip shows a surgical scar overlying a large tumor mass. (**b**,**c**) Pelvic computed tomography (CT) with a three-dimensional reconstruction and magnetic resonance imaging (MRI) revealed a calcified left hip mass infiltrating into the pelvis.

The patient’s sister also had similar masses on her hip (Figure 1) and feet; these masses were excised but recurred post-operatively. No ectopic calcification foci were identified in their parents. The serum phosphate concentrations of the father and mother were normal (Table 1). Both parents were healthy and did not have a history of autoimmune diseases or similar disorders. There was no consanguineous marriage or white or black genetic admixture in this family for the past three generations.

**Figure 2:**
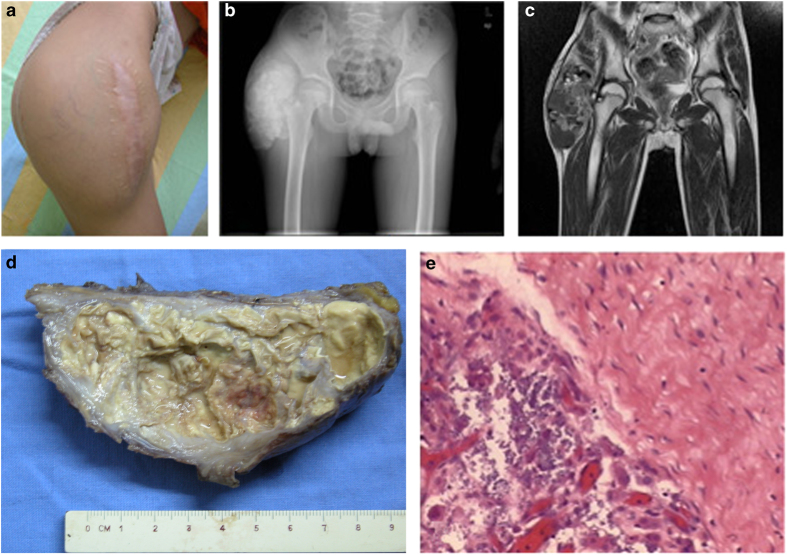
Imaging data of the patient. (**a**) On the right side of the buttocks of the patient, a 14-cm surgical scar with varicosity of the superficial veins in the region was observed in the region of the abnormal mass that was approximately 18 cm in length. (**b**) A computed tomography (CT) reconstruction film of the pelvis shows a calcified soft tissue mass in the right hip. Femur: normal bone density. (**c**) In a T1-weighted magnetic resonance imaging of the pelvis, low intensity signals were obtained at the site of the lesion, indicating the presence of a soft tissue mass in the right hip. (**d**) A 16×3×5 cm tumor specimen was obtained during the operation; the tumor section showed multiple lobes and a gray allantoic fluid. The size of the cyst within the tumor was 1.5×2.5 cm, and the cyst was encapsulated with a 0.5-cm-thick smooth wall and contained a lime-like material. (**e**) Histological analysis showed fibrous tissue hyperplasia of the capsular wall with calcification and giant cell reaction.

Upon physical examination, the proband was conscious and cooperative. Claudication of the right leg was observed. A large, hard mass with slight tenderness was found on the right hip (Figure 2a). The serum phosphate level for this patient was as high as 2.88 mmol·L^−1^ (normal range: 0.8–1.6 mmol·L^−1^). His serum levels of parathyroid hormone (PTH) and 25-hydroxyvitamin D_3_ were normal.

A plain X-ray of the pelvis revealed calcification of a soft tissue mass in the right hip. No cortical hyperostosis, diaphysitis, or periosteal apposition was observed at the femur (Figure 2b) or ankle joints. Magnetic resonance imaging of the pelvis confirmed the findings of the plain radiography (Figure 2c).

An operation to remove the mass on the right hip of the proband was performed in our hospital. The tumor size was ~16×3×5 cm^3^ with multiple lobes and gray allantoic fluid (Figure 2d). The size of the cyst within the tumor was 1.5×2.5 cm^2^, and the cyst was encapsulated by a 0.5 cm thick, smooth wall and contained a lime-like material. The histopathological examination of the tumor mass revealed fibrous tissue hyperplasia within the cyst wall with calcification and giant cell reaction (Figure 2e).

### Genetic analysis

Whole-blood samples were collected from the proband, his sister and family members (9 samples) after written informed consent was obtained. Genomic DNA was isolated from the leukocytes. A direct sequencing method was used to sequence the *GALNT3* and *FGF23* genes of the patients and their relatives (ABI 3700 automated fluorescent sequencer was used for bidirectional sequencing). DNA from another 100 unrelated normal subjects was also tested for these two genes. The potential for DNA sequence alterations and the effects of such alterations on the structure and function of the DNA were evaluated by the online programs MutationTaster^9^ (http:www.mutationtaster.org), PolyPhen-2^10^ (http://genetics.bwh.harvard.edu/pph2) and SIFT^11^ (http://sift.jcvi.org).

## Results

**Figure 3:**
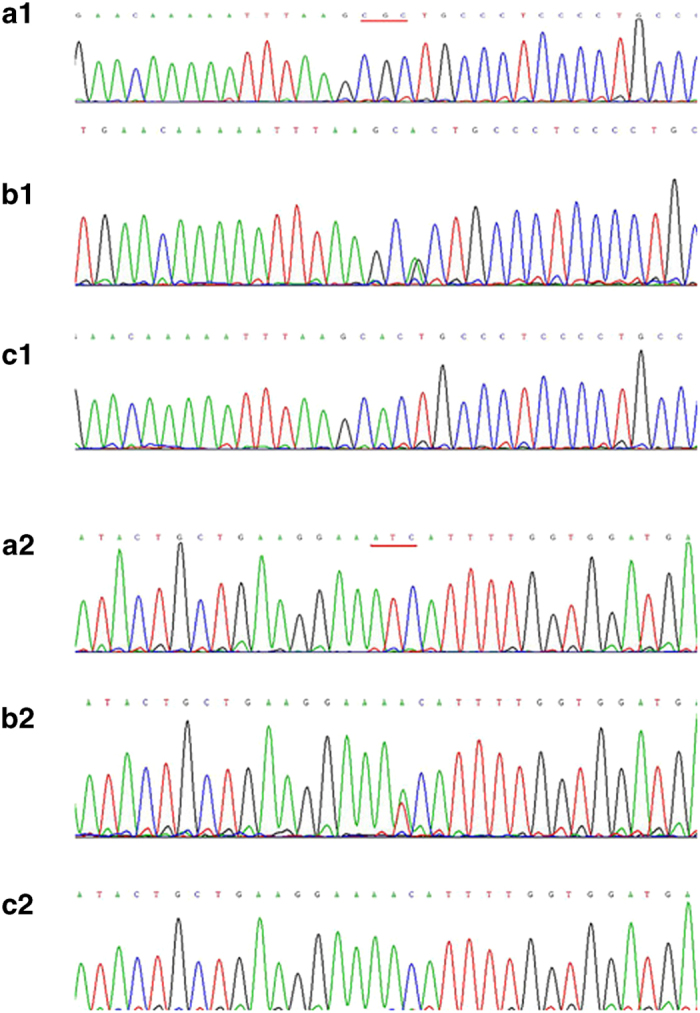
Mutation types of *GALNT3*. The sequence of the PCR-amplified exon 2 of the*GALNT3* gene. Wild-type 539G-A (a1), heterozygous mutation (b1), homozygous mutation (c1). Wild-type 659T-A (a2), heterozygous mutation (b2), homozygous mutation (c2).

As shown in Figure 3, exon 2 of the *GALNT3* gene in both the proband and his sister had 2 homozygous missense mutations: a 539G-A homozygous mutation (Figure 3c1), which resulted in the substitution of arginine 180 with histidine (R180H), and a 659T-A homozygous mutation (Figure 3c2), leading to the substitution of isoleucine 220 with asparagine (I220N). Their parents did not present any clinical characteristics of FTC, but both had the heterozygous mutations of *GALNT3* in exon2 (R180H and I220N; Figure 3b1 and b2). The identified nucleotide changes were not found in other members of the family (Figure 3a1). As predicted by MutationTaster and PolyPhen-2, both of these mutations were “Disease causing”. The results derived from the SIFT analysis also showed that the mutation of I220N is “Damaging” and that R180H is “Tolerated”.

The *FGF23* mutation was not found in the proband or other members of the family. No mutations at these loci were identified in an additional 100 unrelated normal subjects.

## Discussion

This study documents the first Chinese family clinically diagnosed with HFTC and confirmed by genetic analysis, with the identification of two new mutations in *GALNT3*.

FTC is an autosomal recessive, genetic metabolic disorder. The most notable clinical manifestation of FTC is ectopic calcification, predominantly at the hip, elbow joints, shoulders, and knees. FTC rarely affects the skin, intervertebral discs, epidural sites, or perineum. There are two subtypes of FTC, hyperphosphatemic (HFTC) and normophosphatemic FTC. Clearly, the two cases presented here are not normophosphatemic FTC. In addition, our patients did not present bone lesions, such as hyperostosis; therefore, hyperostosis-hyperphosphatemia syndrome, which also results from mutations in the *GALNT3* gene,^1^ could be excluded.

Generally, HFTC occurs before the age of 20. Serum phosphate levels in these patients are elevated, but their circulating calcium and PTH concentrations are normal. The serum levels of 1,25-dihydroxyvitamin D_3_ are normal or slightly higher than normal in these patients. The renal function is normal in these patients.^12,13^

The imaging features of FTC are typical. These tumor masses are often located in the musculus extensor lateralis. Computed tomography typically reveals lobulated calcific masses within the soft tissues with a separate encapsulated cystic component. T1-weighted images from the magnetic resonance imaging show a non-homogeneous low-signal region, whereas the T2-weighted image shows a high-signal region of nodules and a non-signal region in the diffuse low-signal region.^14^ The two patients we presented also had typical imaging features of FTC.

A differential diagnosis of FTC should be made because several other medical conditions may appear similar to FTC in the imaging tests,^1^ such as renal inadequacy, heterotopic calcification secondary to primary hyperparathyroidism, and vitamin D poisoning. Systemic diseases with clinical manifestations of ectopic calcification include mixed connective tissue disease, dermatomyositis, polymyositis, and systemic lupus erythematosus. Neoplastic diseases (including synovial sarcoma, osteosarcoma, and chondrosarcoma) and some degenerative disorders, such as calcified tendonitis, may also have similar radiological findings to those of FTC.^14^ If the patient has no history of the above-mentioned diseases and has a normal renal function, normal serum PTH, and 25-hydroxyvitamin D_3_ levels, heterotopic calcification secondary to the other diseases is unlikely.

The most common molecular pathogenesis of HFTC is related to loss-of-function mutations in *GALNT3,*^1^ which encodes UDP-GalNAc transferase 3 (GalNAc-T3) and mediates the glycosylation of FGF23, a potent phosphaturic protein. In the presence of a *GALNT3* mutation, FGF23 proteins cannot be glycosylated and are vulnerable to degradation, resulting in hyperphosphatemia and FTC.^5,15,16^

*GALNT3* mutations in FTC can occur at various loci within the gene, and there are no obvious hotspot mutations.^17–24^ In our study, both the proband and his sister carried 2 homozygous mutations (R180H and I220N) on exon 2 of the *GALNT3* gene. It has been reported that most *GALNT3* mutations can lead to early termination of protein translation or the defective translation of exons caused by an error in intron splicing.^18^ Mutational analysis using online software programs revealed that these two mutations are both located within the coding region and may cause alterations in the amino acid sequence, which might affect protein features. Thus, these mutations are predicted to be pathogenic. In addition, the mutation I220N within the glycosyl transferase domain may cause splice-site changes. In addition, the novel mutations we identified in exon 2 are adjacent to several previous reported mutations, further suggesting that this region of the* GALNT3* may be susceptible to an increased mutation rate.

The clinical phenotype and severity of HFTC are not always related to the genotype. In our cohort, each patient harbored two homogenous mutations in exon 2 of the *GALNT3* gene and demonstrated severe clinical features, whereas other HFTC patients carrying one homozygous mutation in *GALNT3,* with mild to severe calcification, need multiple operations.^19^ Indeed, the phenotypes of FTC are heterogeneous, with a wide variation in disease severity and involved tissues.^25,26^ For example, Ichikawa S. et al. ^27^described an FTC patient with a compound heterozygote for two mutations in exon 3 and exon 5 of the *GALNT3* gene, resulting in coding changes within the catalytic domain of GalNAc-T3. However, this patient demonstrated only a small eyelid ectopic calcification. In another two symptomatic siblings with compound heterozygous *GALNT3* mutations in exon 4 and 5, one exhibited features of FTC but the other demonstrated hyperostosis-hyperphosphatemia syndrome.^26^ Different *GALNT3* mutations have been reported in the literature (Table 2). It is noteworthy that our two cases are the only ones carrying homozygous mutations. The parents of the patients in our study carried compound heterozygotes for the two mutations in exon 2, which were reported in 11 of 32 families with FTC or hyperostosis-hyperphosphatemia syndrome caused by *GALNT3* mutations (Table 2), but they were reluctant to participate in additional examinations to exclude mild FTC or hyperostosis-hyperphosphatemia syndrome.

Our study has several limitations. Serum FGF23 levels were not measured, and a genetic analysis was not performed on the* KL* gene. In addition, a functional analysis of the *GALNT3* gene was not conducted.

In summary, we identified two novel mutations (R180H and I220N) in exon 2 of the *GALNT3* gene. This report provides the first clinical and genetic description of HFTC in a Chinese family. Further study is needed to explore the mechanism of such a mutation on FGF23 activity. Our findings may assist not only in the clinical diagnosis of HFTC but also in the interpretation of the genetic information used for prenatal diagnosis and genetic counseling.

## Figures and Tables

**Table 1:** The serum biochemical parameters of the current familial tumoral calcinosis (FTC) family

Biochemical parameters	Proband	Sister	Mother	Father
Phosphate/(mmol·L^−1^)	2.88 (0.8–1.6)	1.82	1.17	1.23
Calcium/(mmol·L^−1^)	2.55 (2–2.75)	2.41	2.36	2.28
PTH/(pg·mL^−1^)	15.4 (15–68.3)	48.7	52	30
25(OH)D/(nmol·L^−1^)	69.2 (44.7–144)	37.5	41.2	52.4
Creatinine/(μmol·L^−1^)	46 (53–115)	53	74	82

**Table 2:** A summary of currently known *GALNT3* mutations and their associated clinical features

Family	Mutation (cDNA)	Mutation (protein)	Exon	Phenotype	Homozygous compound heterozygotes	References
1	*c.2T>A*	p.M1?	1	Hyperostosis		^20^
	*c.839G>A*	p.C280Y	4		Compound heterozygotes	
2	*c.842A>G*	p.E281G	4	Tumoral calcinosis, hyperostosis		^26^
	*c.1097T>G*	p.L366R	5		Compound heterozygotes	
3	*c.1312C>T*	p.R438C	6	Hyperostosis, tumoral calcinosis, thyroid		^28^
	*c.1774C>T*	p.Q592X	9	calcification and other atypical symptoms	Compound heterozygotes	
4	*c.516-2A>T*	p.C173VfsX4	2	Tumoral calcinosis		^21^
5	*c.1313G>A*	p.R438H	6	Hyperostosis	Homozygous	^21^
6	*c.1102_1103insT*	p.S368FfsX8	5	Tumoral calcinosis	Homozygous	^18^
7	*c.1460G>A*	p.W487X	7	Tumoral calcinosis	Homozygous	^18^
8	*c.966T>G*	p.Y322X	4	Tumoral calcinosis	Homozygous	^16^
	*c.1441CT*	p.Q481X	7		Compound heterozygotes	
9	*c.803-804insC*	p.A268fsX4	3	Hyperostosis		^5^
	*c.1626+1G>A*	splice site	8		Compound heterozygotes	
10	*c.815C>A*	p.T272K	3	Tumoral calcinosis		^27^
	*c.1076C>A*	p.T359K	5		Compound heterozygotes	
11	*c.41_58del*	p.R14fs21X	1	Tumoral calcinosis		^22^
12	*c.1387A>T*	p.K463X	6	Tumoral calcinosis	Homozygous	^12^
13	*c.1774C>T*	p.Q592X	9	Tumoral calcinosis	Homozygous	^23^
14	*c.484C>T c.516-2A>T*	p.R162X p.C173VfsX4	1	Tumoral calcinosis	Homozygous	^9^
15	*c.1524+1G>A*	p.K465_Y508del	7	Hyperostosis	Compound heterozygotes	^23^
16	*c.1524+1G>A*	p.K465_Y508del	7	Tumoral calcinosis		^3^
17	*c.1524+5G>A*	Splice site	7	Tumoral calcinosis	Homozygous	^3^
	*c.484C>T*	p.R162X	1		Homozygous	
18	*c.485G>A*	p.R162Q	1	Tumoral calcinosis	Compound heterozygotes	^17^
19	*c.1392+1G>A*	Splice error	6	Hyperostosis		^17^
20	*c.677delC*	p.A226VfsX3	2	Tumoral calcinosis	Homozygous	^17^
21	*c.1720T>G*	p.C574G	9	Tumoral calcinosis	Homozygous	^17^
22	*c.1245T>A*	p.H415Q	6	Tumoral calcinosis	Homozygous	^19^
23	*c.1312T>C*	p.R438C	6	Tumoral calcinosis	Homozygous	^19^
24	*c.1524+1G>A*	p.K465_Y508del	7	Hyperostosis	Homozygous	^20^
25	*c.767G>T*	p.G256V	3	Tumoral calcinosis	Homozygous	^21^
26	*c.484C>T*	p.R162X	1	Tumoral calcinosis	Homozygous	^23^
27	*c.484C>T*	p.R162X	1	Tumoral calcinosis	Homozygous	^24^
28	*c.516-2A>T*	p.C173VfsX4	2	Tumoral calcinosis	Homozygous	^22^
	*c.260-266del*	p.R87QfsX2	1		Homozygous	
29	*c.1584-1585insA*	p.P529TfsX17	8	Tumoral calcinosis	compound heterozygotes	^22^
30	*c.516-2A>T*	p.C173VfsX4	2	Tumoral calcinosis		^22^
	*c.1524+5G>A*	Splice site	7		Homozygous	
31	*c.746-749del*	p.R250TfsX2	3	Tumoral calcinosis	Compound heterozygotes	^22^
	*c.892del*	p.Y298SfsX5	4			
32	*c.539G>A*	p.R180H	2	Tumoral calcinosis	Compound homozygous	Present study
	*c.659T>A*	p.I220N	2	Tumoral calcinosis		Present study
